# Economic Welfare, Food Prices, and Sectoral Food Waste: A Structural Analysis Across the European Union

**DOI:** 10.3390/foods15020403

**Published:** 2026-01-22

**Authors:** Anca Antoaneta Vărzaru

**Affiliations:** Department of Economics, Accounting and International Business, University of Craiova, 200585 Craiova, Romania; anca.varzaru@edu.ucv.ro; Tel.: +40-7-7392-1189

**Keywords:** food waste, primary production, manufacturing, retail, food services, households, European Union

## Abstract

Food waste remains a significant challenge in the European Union, reflecting structural differences across economic sectors and member states. This study examines how macroeconomic conditions relate to sectoral food waste using harmonized Eurostat data for the EU-27, covering five stages of the food chain and three economic indicators: GDP (Gross Domestic Product) per capita, adjusted gross disposable income per capita, and the Harmonized Index of Consumer Prices for food. The research design integrates factor analysis, structural equation modeling, and hierarchical clustering. Results show that income-related variables have a positive, statistically significant effect on overall food waste, particularly in manufacturing and distribution. In contrast, food prices show a negative, statistically non-significant relationship with waste generation. Cluster analysis identifies two statistically distinct country groups; however, substantial internal heterogeneity indicates that these clusters reflect structural economic configurations rather than typological or behavioral categories. The findings suggest that macroeconomic factors partially explain cross-country differences in food waste and support the need for context-sensitive, sector-specific policy interventions.

## 1. Introduction

Food waste has become a significant challenge for modern food systems, highlighting economic inefficiencies, environmental concerns, and social inequalities throughout the agri-food chain. In the European Union (EU), this problem has gained increasing academic and policy focus due to its effects on sustainability, resource efficiency, and food security [1,2,3]. Evidence indicates that food losses and waste happen at every stage of the value chain, from primary production to household consumption, impacting not only resource depletion but also climate change and economic stability [4,5]. Nonetheless, food waste levels vary widely across EU countries, reflecting the Union’s diverse structural, economic, and institutional contexts [6,7].

Research indicates that food waste results from a complex interplay of behavioral, technological, and macroeconomic factors. Economic prosperity, food prices, and purchasing power shape consumption habits, production levels, and tolerance for inefficiencies throughout the food supply chain [8,9]. Wealthier regions tend to generate more food waste due to higher consumption, diverse diets, and greater price insensitivity, while lower-income areas often struggle with infrastructure, storage, and logistical issues [10,11]. These patterns mirror broader socioeconomic trends influencing production and consumption across Europe [12,13,14]. Despite extensive research, limited empirical data exist on how macroeconomic indicators directly affect sector-specific food waste outcomes across the EU.

This research addresses a gap by examining how key macroeconomic indicators—GDP per capita, adjusted gross disposable income per capita, and food price trends—are associated with food waste across five stages of the food supply chain: primary production, manufacturing, retail and distribution, food services, and households. While earlier studies have primarily focused on behavioral, cultural, or household factors influencing food waste [15,16,17,18,19], few have combined sector-specific food waste data with standardized macroeconomic indicators across Europe. Furthermore, comparative analyses such as those by Szulecka et al. [20] and Giordano et al. [21] often examine individual countries or governance structures rather than multiple countries within a unified structural framework.

The analysis posits that macroeconomic factors shape production, market structures, and consumer habits, which in turn differentially affect food waste generation across sectors. Income indicators determine purchasing power, consumption levels, and tolerance for inefficiency, particularly in the late and middle stages of the food supply chain. Meanwhile, food prices affect the opportunity cost of waste. These interactions are shaped by sector-specific traits, technological levels, and institutional settings, leading to varied patterns of food waste across EU countries.

This study contributes by reframing the established relationships among income, prices, and food waste from a sectoral and structural perspective at the European Union level. Rather than treating food waste primarily as a household or aggregate issue, the analysis shows that macroeconomic effects are unevenly distributed across the food supply chain. Income-related factors are concentrated in the manufacturing and retail–distribution stages, while their influence is weaker or unclear in primary production and households. The study also interprets cross-country similarities not as driven by behavioral or cultural factors, but as structural patterns linked to income distribution, price fluctuations, and sector-specific waste profiles. Using factor analysis, structural equation modeling, and clustering, the paper identifies where macroeconomic explanations are most applicable and where their explanatory power is limited. This approach builds on prior work on tailored, context-sensitive policies [22,23] and provides a structural foundation for aligning food waste reduction efforts with broader sustainability and circular economy goals [8,24,25].

Using macroeconomic indicators and cross-country food waste data is limited by variations in national waste measurement methods and statistical practices, which can undermine international comparability. These limitations are explicitly acknowledged in the analysis of the empirical results and underscore the importance of a structural approach rather than a purely typological comparison of countries.

The paper is organized as follows: The next section reviews the theoretical and empirical literature on factors influencing food waste in the European Union. The Materials and Methods section outlines the data sources and analytical techniques. Subsequent sections present and interpret the empirical results. The paper concludes with a summary of key findings, policy implications, and recommendations for future research.

## 2. Theoretical Background

### 2.1. Economic and Behavioral Factors Influencing Food Waste Generation in the European Union

Food waste has increasingly been conceptualized in the European research literature as a structural outcome of imbalances among production capacity, market organization, and consumption behavior. In line with FAO [2] and Cattaneo et al. [1], food waste reflects systemic inefficiencies within the agri-food system, with direct implications for sustainability and food security. The heterogeneity observed across EU member states indicates that food waste is not merely a behavioral issue but is deeply embedded in economic structures and sector-specific dynamics along the food supply chain.

From a macroeconomic perspective, food waste generation is closely linked to income levels, purchasing power, and food price dynamics [8,9]. Higher income levels typically correlate with lower price elasticity and increased consumption, which results in more waste, particularly from a supply chain perspective. Empirical studies show that wealthier economies tend to generate more food waste at the consumption stage, while lower-income or emerging economies experience relatively higher losses during production and processing [26,27]. This divergence reflects differences in market structure, technological focus, and supply chain setup, rather than being directly driven by consumption patterns [10,28,29].

Economic modeling approaches developed by Rutten et al. [30,31] and Campoy-Muñoz et al. [32] provide further theoretical insight into these mechanisms. Their results indicate that reducing food waste can improve resource efficiency and food security, but may also lead to adverse outcomes, such as reduced agricultural output and employment [33]. These findings underscore that food waste is not only a moral or environmental issue but also a macroeconomic phenomenon shaped by production scale, income distribution, and market incentives.

Supply-side factors further mediate the relationship between macroeconomic conditions and food waste. Bioeconomic research using the MAGNET model [8] highlights the roles of logistics, packaging, and labeling in shaping waste outcomes. Packaging, in particular, influences both purchasing decisions and disposal behavior, thereby linking market strategies to household waste generation [34,35]. Behavioral research based on the theory of planned behavior highlights attitudes, social norms, and perceived control as critical explanatory factors; however, in this analysis, they are cited only as contextual insights from the literature and are not directly measured [36,37]. Studies by Attiq et al. [15] and Kansal et al. [38] show that these behavioral mechanisms interact with economic conditions, producing distinct waste patterns across sectors and countries.

Cross-country empirical evidence highlights substantial variation in food waste levels among EU member states [6,39]. These differences are partly attributable to measurement inconsistencies but also reflect more profound economic and institutional disparities [5,11]. As Corrado and Sala [11] and Scherhaufer et al. [5] emphasize, harmonizing waste accounting systems is essential for accurately linking macroeconomic indicators to sectoral food waste. The persistence of sector-specific variation suggests that economic context and institutional capacity jointly shape waste outcomes [7,40].

Economic welfare plays a central role in explaining these disparities. Rising purchasing power and declining relative food prices tend to lower the perceived value of food, increasing the likelihood of waste, particularly at the household level [9,41]. At the same time, regional development, infrastructure quality, and food education significantly condition how income and prices translate into waste generation [2,42]. Empirical studies confirm that income levels and household characteristics are key determinants of waste quantities [43,44], although targeted awareness campaigns and behavioral interventions can partially offset these effects [45,46,47].

Overall, the literature indicates that income-related indicators are structurally associated with higher levels of food waste, particularly in the downstream and intermediate sectors of the food supply chain, without directly observing individual consumption decisions. In contrast, food prices are theoretically expected to reduce food waste by raising the opportunity cost of waste. However, the magnitude of these effects is likely to vary across sectors and socioeconomic contexts [1,23,48,49,50]. On this basis, the following hypothesis is formulated:

**Hypothesis H1.** 
*Variations in economic welfare indicators and food prices are systematically associated with food waste generation across sectors, with income expected to increase food waste and higher food prices expected to reduce it.*

This study adopts a structural economic perspective, and any references to behavioral mechanisms serve solely to support theoretical interpretation rather than to infer empirically observed behavior.

### 2.2. Socioeconomic Typologies and Institutional Convergences: Foundations for Grouping EU Member States

Beyond individual economic drivers, food waste patterns are shaped by institutional arrangements and governance models. Comparative studies by Szulecka et al. [20] show how administrative traditions and policy frameworks produce distinct food waste management approaches across countries. France’s regulatory, top-down model [51], Italy’s cooperative, network-based approach [21,52], the United Kingdom’s reliance on voluntary corporate initiatives [53], and Norway’s sectoral partnership model [54] demonstrate how governance structures interact with economic conditions.

Recent research indicates that EU food waste policies increasingly combine regulatory instruments, economic incentives, and voluntary measures [55,56]. While this hybrid governance model may enhance flexibility and stakeholder involvement, it can also obscure underlying structural drivers of food waste [57,58]. Analyses of multi-actor governance show that EU member states can be grouped into relatively stable institutional configurations, shaped by political commitment, economic maturity, and social capacity [20,59].

Methodologically, cluster analysis has been widely used to classify countries based on economic and environmental indicators [60,61], with more recent studies advocating multidimensional approaches that integrate economic, behavioral, and institutional variables [62,63]. Such approaches support the argument that food waste profiles are structurally determined and cannot be reduced to isolated behavioral patterns. Priefer et al. [64] emphasize the importance of tailoring public policy to empirically identified waste profiles, while Göbel et al. [65] highlight value-chain cooperation as a key determinant of national waste outcomes.

Clustering techniques thus provide an analytical tool for capturing structural similarities and differences among EU member states. Concepts such as “institutional ecologies” describe how interactions among markets, regulation, and civil society generate distinct waste trajectories [66,67]. Empirical evidence further confirms that food waste is closely linked to broader socioeconomic structures and environmental pressures [8,68,69]. Accordingly, the following hypothesis is proposed:

**Hypothesis H2.** 
*European Union member states can be grouped into statistically distinct clusters based on their sectoral food waste profiles and socioeconomic indicators, reflecting structural economic and institutional configurations rather than uniform behavioral typologies.*

## 3. Materials and Methods

### 3.1. Research Design

The research design of this study is grounded in the view that food waste generation in the European Union is a complex, multifaceted issue shaped by the interplay of economic structures, consumption habits, and sectoral behaviors. The study employs both explanatory and exploratory approaches, combining quantitative modeling with comparative typology, to understand not only the extent of food waste but also its underlying structural causes. Guided by the hypotheses that differences in macroeconomic indicators can explain sectoral diversity and that EU member states can be grouped into similar clusters based on their waste production and socioeconomic characteristics, the design integrates descriptive, inferential, and predictive methods into a unified analytical framework.

The conceptual framework links food waste across five economic sectors: primary production, manufacturing, retail and distribution, restaurants and food services, and households, with three key macroeconomic indicators: GDP per capita, the Harmonized Index of Consumer Prices for food, and adjusted gross disposable income per capita. By situating these variables within the broader European context, the design captures the interplay among market trends, consumption capacity, and production efficiency.

The study follows a sequential structure: it first applies factor analysis to reduce dimensionality and identify interdependencies among variables, then uses structural equation modeling, and finally conducts cluster analysis to group countries with similar waste and economic patterns. This integrated research approach enables the investigation to go beyond basic statistics, offering a detailed account of how economic well-being and structural differences shape the distribution of food waste across the EU.

The analysis focuses on 2020–2022, the latest period for which complete and harmonized Eurostat data on sectoral food waste and macroeconomic indicators are available for all EU-27 countries. Earlier years are limited due to data gaps and inconsistent methods, reducing comparability across countries. This timeframe aligns with the COVID-19 pandemic, which is viewed as a standard external shock affecting all EU member states, enabling structural cross-country comparisons based on common external factors rather than analyzing specific behavioral changes caused by the pandemic.

### 3.2. Selected Variables

The empirical foundation of this study is secondary data from Eurostat, the most recent and standardized series available for the 27 member states of the European Union (2020–2022). The dataset was designed to capture both the extent and structure of food waste, as well as the socioeconomic context in which it occurs. All data were converted to kilograms per person or to purchasing power standards to ensure comparability across countries and sectors.

The dependent variables include indicators of food waste per person across all stages of the food supply chain: losses from agriculture, fishing, and aquaculture; waste produced during industrial processing; waste resulting from inefficiencies in distribution and storage; losses associated with food preparation and service activities; and domestic waste, which remains one of the most significant contributors to overall losses.

The independent variables were chosen to reflect the macroeconomic environment influencing waste generation. GDP per capita (GDPpc), expressed as a percentage of the EU27 average based on purchasing power standards, serves as a proxy for national economic development and consumption capacity. The Harmonized Index of Consumer Prices for Food (HICP), with 2015 as the base year, captures price trends in the food sector and their effects on purchasing and disposal behaviors. Lastly, adjusted gross disposable income per capita (AGDIpc) indicates households’ real spending power, linking economic well-being to consumption and waste patterns.

The dataset was checked for missing or inconsistent data before analysis. Observations with incomplete information were removed to maintain consistency across variables and countries, leading to a balanced dataset for all EU-27 members. No data imputation was used to prevent potential bias from estimation. Table 1 exposes the research variables.

Variable selection was based on both theoretical relevance and data availability. We chose GDP per capita and adjusted gross disposable income to represent economic development and household purchasing power. The Harmonized Index of Consumer Prices for food was selected to indicate price trends influencing consumption and waste behavior. Sectoral food waste indicators were included to cover all key stages of the food supply chain for a thorough structural analysis.

Though Eurostat data are harmonized across the EU, cross-country comparability is limited by differences in national measurement practices, sectoral coverage, and estimation techniques. Notably, food waste from households and food service establishments may be underestimated due to reliance on surveys or indirect reporting. These limitations were taken into account when analyzing differences between countries and sectors.

### 3.3. Methods

The empirical strategy follows sequential, multi-method design. Factor analysis is first applied to identify latent structures and shared variance across sectoral food waste indicators and macroeconomic variables. Structural equation modeling is then used to estimate directional relationships among latent constructs, enabling a simultaneous assessment of the effects of income and price on food waste. Finally, cluster analysis is employed to group EU member states based on standardized scores, translating multivariate similarities into structural groupings. Each method addresses a distinct analytical objective and builds on the results of the previous stage.

Before conducting factor analysis and structural equation modeling, all variables were standardized using z-scores to account for differences in measurement units and scales. This step ensures statistical comparability and prevents variables with larger variances from disproportionately influencing the results. No additional transformations were applied, as distributional diagnostics indicated acceptable levels of skewness and kurtosis.

The first stage involves factor analysis, a multivariate statistical method used to identify latent relationships among correlated variables. By reducing the original dataset to a smaller set of unobserved factors, FA identifies the underlying dimensions that explain most of the data’s variance [74].

The equation can be expressed as follows:

(1) X=AF + ∈

X—observed variables;
A—factor loadings;
F—factors;
∈—errors.

This stage uncovers the relationships between food waste indicators and macroeconomic conditions, offering a condensed view of the system’s structure.

The second analytical stage uses structural equation modeling (SEM), which allows for simultaneous testing of relationships among variables. The general structural form can be written as Equation (2):

(2) $$\eta_{i}=\alpha_{\eta }+B\eta_{i}+\Gamma \xi_{i}+\zeta_{i}$$

*η*, *ξ*—endogenous and exogenous variable vectors;
*B*—effects of the latent endogenous variables on each other;
*Γ*—effects of the latent exogenous variables on the latent endogenous variables;
*ζ*—disturbances;
*i*—cases.

The model repeatedly reduces the prediction error through backpropagation, enabling it to approximate the functional relationship between macroeconomic indicators and sectoral food waste generation [75].

Finally, cluster analysis is used to group EU member states into homogeneous categories based on their combined waste and economic profiles. Hierarchical clustering, employing average linkage and Euclidean distance, establishes initial group structures, which are then refined through k-means clustering to enhance within-cluster similarity [76]. The most suitable method applied was the average linkage approach between groups, expressed as follows:

(3) $$d_{ij}=\frac{1}{kl}\sum_{i=1}^{k}\sum_{j=1}^{l}d(X_{i},Y_{j})$$

$X_{1},X_{2},\ldots ,X_{k}$—observations from cluster 1;
$Y_{1},Y_{2},\ldots ,Y_{l}$—observations from cluster 2;
*d*(*X*,*Y*)—distance between subjects;
*k*, *l*—cases.

The number of clusters was determined by examining the agglomeration schedule and dendrogram, focusing on significant increases in fusion coefficients that indicate meaningful structural separation. A two-cluster solution was retained because it provided the most interpretable balance between statistical distinction and analytical parsimony. Cluster stability was assessed by comparing hierarchical and k-means solutions, which produced consistent group structures.

This sequential application of methods, including factor extraction, structural equation modeling, and clustering, enables both dimensionality reduction and interpretive depth. Factor analysis reveals structural dependencies; structural equation modeling identifies relationships; and the clustering approach translates these insights into meaningful typologies. The combined methodology thus integrates statistical rigor with exploratory flexibility, providing a unified framework for understanding how macroeconomic variability shapes sectoral food waste patterns and the typological segmentation of EU member states.

## 4. Results

### 4.1. Factorial Analysis

The results of the factor analysis of variables describing food waste generation and the macroeconomic context of European Union member states summarize the relationships among these factors. Before factor extraction, the data’s suitability was assessed using standard diagnostic tests. The Kaiser–Meyer–Olkin (KMO) measure of sampling adequacy yielded a value of 0.697, indicating a moderate but acceptable level of sampling adequacy for factor analysis. Bartlett’s test of sphericity was statistically significant (*χ*^2^ = 173.796, *p* < 0.001), indicating sufficient correlations among the variables. Together, these diagnostics support the application of factor analysis to the dataset. The determinant of the correlation matrix (0.103) suggests that collinearity is not severe, allowing for the extraction of clear common factors (Table A1 in Appendix A).

The correlation matrix shows several significant links between sectoral food waste variables and economic indicators. A strong positive correlation is found between food waste in retail and distribution (RODF) and in food manufacturing (MFPB) (r = 0.501, *p* < 0.01), highlighting a structural link between the middle stages of the agri-food chain. GDP per capita (GDPpc) is strongly positively correlated with adjusted gross disposable income (AGDIpc) (r = 0.710, *p* < 0.01) and negatively correlated with the Harmonized Index of Consumer Prices for food (HICP) (r = −0.389, *p* < 0.01), indicating that higher economic development levels are associated with lower inflationary pressures on food prices.

Methodologically, the principal axis factoring method produced a single factor with an eigenvalue of 2.512, explaining 51.4% of the total variance and, after adjustment, 42.77% of the variance. Although moderate, this amount of explained variance is sufficient to identify a latent dimension combining the economic and behavioral determinants of food waste.

The factor matrix shows significant loadings for Manufacture of Food Products and Beverages (MFPB) (0.619), Retail and Other Distribution of Food (RODF) (0.673), and GDP per capita (0.503), along with a negative contribution from the food price index (HICP = −0.552) (Table 2).

Overall, the factor analysis identifies a single dominant latent dimension linking macroeconomic conditions to sectoral food waste. This factor is primarily driven by food waste in manufacturing, retail, and distribution, as well as by income-related indicators (GDP per capita and adjusted gross disposable income). In contrast, food prices load negatively on the same dimension. Food waste in primary production and households shows weaker associations with the extracted factor. These results indicate a clear differentiation between income-related effects, which are structurally aligned with higher waste levels in intermediate supply-chain stages, and price-related effects, which operate in the opposite direction but contribute less to the overall variance captured by the factor.

The weak loadings seen for primary production and household food waste imply that these stages are less directly connected to the main macroeconomic factor identified. This suggests sector-specific dynamics that go beyond general income and price influences.

Given the moderate KMO value and the explained variance, factor analysis is used here as an exploratory tool to identify key structural dimensions linking macroeconomic indicators to sectoral food waste. The goal is not to develop a complete latent variable model.

### 4.2. Structural Equation Modeling

Partial least squares SEM was chosen because it is well-suited for exploratory analysis, effectively handles moderate sample sizes, and aims to maximize explained variance, especially when theoretical relationships are complex and not yet fully established.

**Figure 1:**
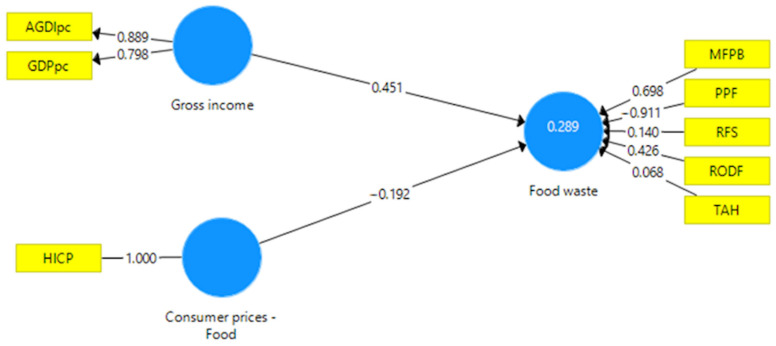
Structural equation model linking macroeconomic indicators and sectoral food waste across EU member states. Source: author’s design with SmartPLS v3.0 (SmartPLS GmbH, Bönningstedt, Germany).

The structural equation model developed for this study was designed to capture the interplay between three latent constructs central to understanding food waste dynamics in the European Union: Consumer Prices—Food, Gross Income, and Food Waste (Figure 1).

The latent construct Consumer Prices—Food was represented by the Harmonized Index of Consumer Prices for food (HICP), which reflects the inflationary context shaping consumer expenditure decisions. Gross Income was measured through two indicators, GDP per capita (GDPpc) and adjusted gross disposable income per capita (AGDIpc), capturing both national economic development and household-level purchasing power. Food Waste incorporated multiple sector-specific indicators, namely MFPB, PPF, RFS, RODF, and TAH, reflecting the multidimensional nature of waste generation across production, processing, distribution, services, and households.

The SEM was estimated using partial least squares, an approach suited for exploratory modeling in contexts where theoretical relationships are complex and data are moderately sized. The model displayed acceptable explanatory power, with an R^2^ of 0.289 for the latent variable Food Waste (adjusted R^2^ = 0.271). Although modest, this level of explained variance is consistent with the multifactorial nature of food waste, which is shaped by economic, behavioral, cultural, and infrastructural factors.

Reliability and validity diagnostics confirmed the model’s internal consistency. Composite reliability was strong for Gross Income (CR = 0.833), and indicator collinearity remained within acceptable bounds, as indicated by VIF (Variation Inflation Factor) values well below the critical thresholds. Measures of model fit, including an SRMR (Standardized Root Mean Squared Residual) of 0.035 and an NFI (Normed Fit Index) of 0.933, indicated a well-fitting model, with saturated and estimated models yielding identical values across fit indices, suggesting overall robustness.

The structural paths offered important insights into the relationships among economic indicators and food waste (Table 3).

The path from Consumer Prices—Food to Food Waste was negative and statistically non-significant (β = −0.214, *p* = 0.173), suggesting that food price fluctuations do not directly translate into changes in waste levels across sectors. This finding shows that in wealthier economies, food price changes have a weaker overall connection with food waste results. This implies that price influences are more structural and less about immediate consumer reactions.

In contrast, the path from Gross Income to Food Waste was positive and statistically significant (β = 0.407, *p* = 0.012). At the structural level, increased purchasing power correlates with overall higher consumption intensity and reduced price elasticity, leading to elevated food waste across multiple sectors. This relationship appears at the system level, encompassing the household and distribution sectors, where increased waste aligns more with income-related structural factors than with direct individual purchasing habits.

The structural equation model explains a meaningful share of variance in Food Waste (R^2^ = 0.289), and bootstrapped R^2^ values remain statistically significant (*p* = 0.001). Model fit diagnostics, including those that fall below their respective upper confidence bounds, confirm the stability of the estimates and the adequacy of the measurement structure. Accordingly, the SEM results are interpreted as indicative of structural associations at the system level rather than as confirmatory evidence of fully specified causal mechanisms.

Regarding the structural paths, Gross Income has a positive, statistically significant effect on Food Waste, whereas the impact of Consumer Prices—Food is negative but not statistically significant. The limited contribution of primary production and household indicators to the Food Waste construct further suggests that macroeconomic drivers exert uneven influence across the food supply chain, with weaker explanatory power in sectors shaped by non-market and informal dynamics.

These results provide differentiated support for Hypothesis H1: income-related indicators emerge as a robust determinant of food waste across sectors, whereas food prices show a weaker, statistically inconclusive association.

### 4.3. Cluster Analysis

The cluster analysis was conducted to identify groups of EU member states that share comparable profiles in terms of sector-specific food waste and macroeconomic characteristics. The study used data from 2022, the most recent year for which harmonized Eurostat data are available for all variables. To ensure comparability across indicators with different units and scales, all variables were standardized using z-scores before computing distances. This step is essential when applying Euclidean metrics, as unstandardized values would distort the clustering structure by over-weighting variables with larger variances.

The clustering procedure employed squared Euclidean distance and the average linkage (between-groups) method. The selected variables were the five sector-specific indicators of food waste—PPF (primary production), MFPB (manufacturing of food and beverages), RODF (retail and distribution), RFS (restaurants and food services), and TAH (households)—together with three macroeconomic variables: GDPpc (GDP per capita in PPS), HICP (Harmonized Index of Consumer Prices for food), and AGDIpc (adjusted gross disposable income per capita in PPS).

**Figure 2:**
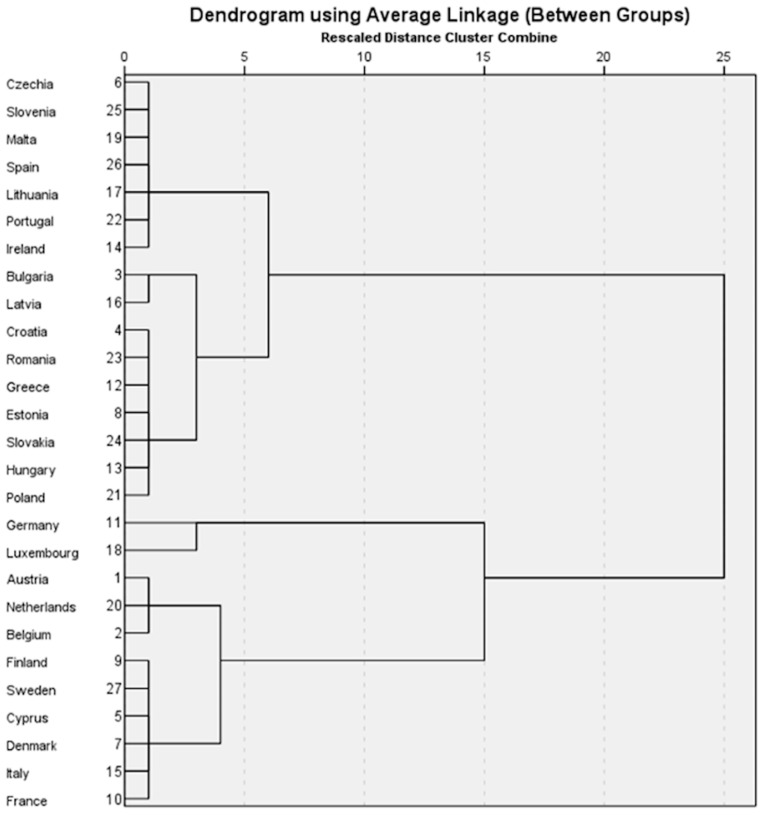
Hierarchical cluster structure of EU member states based on sectoral food waste and macroeconomic indicators (2022). Source: author’s design using SPSS v.27 (IBM Corporation, Armonk, NY, USA).

To determine the number of clusters, the agglomeration schedule and dendrogram were examined jointly (Figure 2 and Table A2 in Appendix A).

The most significant increase in the agglomeration coefficient occurred at the step where two large, distinct groups merged, providing empirical support for a two-cluster solution. While no silhouette or elbow statistics are available for hierarchical clustering with average linkage, the noticeable jump in fusion coefficients and the clear bifurcation of the dendrogram provided sufficient justification to retain a two-cluster structure. The resulting clusters represent statistical groupings and should not be interpreted as culturally or behaviorally homogeneous categories. While the two-cluster solution is supported by consistency between hierarchical and k-means procedures, the resulting clusters should be interpreted as robust statistical groupings based on multivariate similarity rather than as definitive behavioral, cultural, or institutional typologies.

The first cluster (Cluster A) comprises countries across Central, Eastern, and parts of Southern Europe. These states tend to have lower GDPpc and AGDIpc and higher HICP than the EU average, though there is notable internal heterogeneity. Within this group, countries such as Czechia, Slovenia, Malta, Spain, Lithuania, Portugal, and Ireland exhibit sectoral food-waste profiles with moderate PPF and MFPB and comparatively higher household (TAH) and food-service (RFS) waste. Others, such as Bulgaria, Latvia, Croatia, Romania, Greece, Estonia, Slovakia, Hungary, and Poland, show higher PPF and MFPB and greater variation in household waste. The internal variation within Cluster A underscores that the grouping is statistical rather than typological and should not be overstated in interpretive terms.

The second cluster (Cluster B) comprises higher-income Western and Northern European economies, including Cyprus. These countries generally have higher GDPpc and AGDIpc and lower or more stable HICP than those in Cluster A. Their sectoral food waste patterns differ markedly from those in Cluster A, with substantially higher mean levels in MFPB and RODF. However, as in the first cluster, internal heterogeneity is substantial. For example, Cyprus and Denmark show very high values across multiple waste categories, whereas countries such as Finland and Sweden are much closer to the EU average. The presence of such internal variability indicates that the cluster solution captures broad structural contrasts but cannot be interpreted as representing uniform behavioral patterns within groups.

Cluster analysis identifies a statistically distinguishable two-cluster structure. Cluster A includes countries with generally lower income levels and heterogeneous sectoral food waste profiles. By comparison, Cluster B comprises higher-income economies with higher average waste levels in manufacturing, retail, and distribution. Despite this statistical separation, substantial internal heterogeneity remains within both clusters.

These results provide partial support for Hypothesis H2. The identified clusters capture broad structural similarities in the joint distribution of macroeconomic indicators and sectoral food waste rather than cohesive economic or cultural types. Accordingly, the cluster solution should be interpreted as an analytical tool summarizing multivariate patterns, not as a definitive typology of EU member states.

The clusters are statistical constructs derived from multivariate similarities and should be interpreted as structural groupings rather than homogeneous economic, cultural, or behavioral types. The observed internal heterogeneity within clusters is therefore an inherent feature of the analytical approach and does not weaken the relevance of the clustering results.

## 5. Discussion

The results highlight an apparent asymmetry in the effects of income and price on food waste generation across the European Union. More importantly, these findings reframe well-known income–food waste relationships within a sectoral and structural context, illustrating that macroeconomic impacts vary across the food system and are primarily concentrated in certain stages of the supply chain. This section discusses these findings in relation to existing theoretical and empirical literature and explores their implications for policy and future research. The results support both working hypotheses, offering a detailed understanding of the connections among the macroeconomic environment, sectoral structure, and food waste behaviors across the European Union. More than just statistical validation, these findings are significant in the context of existing research, which consistently emphasizes the complex, contextual nature of food waste.

With respect to Hypothesis H1, the results show that macroeconomic conditions are associated with food waste generation in a differentiated manner. Income-related variables show a positive, statistically significant association with overall food waste, consistent with previous studies linking higher economic welfare to increased consumption intensity and system-level inefficiencies [8,9]. These findings align with evidence that, in high-income contexts, food expenditure accounts for a relatively small share of household budgets, thereby reducing the constraining role of prices on aggregate waste outcomes.

By contrast, food prices, as measured by the HICP, do not exhibit a statistically significant direct association with food waste. This distinguishes the present results from studies conducted in lower-income or non-European contexts, where price effects are often stronger. The findings suggest that, within the EU, price-based mechanisms alone provide limited explanatory power for food waste patterns, supporting arguments that income-related structural conditions play a more prominent role in economically advanced and diverse regions [8,9].

Sectoral analysis further refines this interpretation. Factor analysis and SEM results indicate that macroeconomic indicators are more closely associated with food waste generated in intermediate stages of the supply chain, particularly manufacturing (MFPB) and retail and distribution (RODF), than with garbage at the household or primary production levels. This pattern is consistent with the work of Rutten et al. [31] and Campoy-Muñoz et al. [32], who emphasize that economic expansion may simultaneously increase food availability while intensifying structural inefficiencies in technologically advanced supply chains.

In contrast, the weaker association between macroeconomic variables and household waste is consistent with literature suggesting that non-economic factors influence household waste; however, these factors are not directly captured in the present structural analysis [15,16,38]. In the case of primary production, the weak association with macroeconomic indicators may reflect sector-specific characteristics such as dependence on weather, biological production cycles, and on-farm management practices, as well as the prevalence of informal losses that are imperfectly captured in official statistics. These features limit the extent to which aggregate income or price indicators can explain waste generation at this stage of the food chain. Similarly, the relatively weak macroeconomic associations observed for household food waste are consistent with evidence that household waste is influenced by factors such as routines, food literacy, storage practices, and social norms, which operate largely independently of aggregate income and price levels [77]. In addition, household food waste measurement relies heavily on surveys and indirect estimation, which may attenuate observable relationships with macroeconomic indicators. Taken together, these comparisons suggest that Hypothesis H1 is supported in a differentiated manner: economic welfare is a key driver of food waste, but sectoral characteristics and non-economic factors mediate its influence.

The 2020–2022 period coincides with the COVID-19 pandemic, which is likely to have affected food waste patterns across sectors. Household food waste may partly reflect increased home cooking, altered shopping habits, and stockpiling. Meanwhile, food waste in restaurants and food services should be understood in the context of temporary closures, capacity limits, and fluctuating demand. These pandemic-related factors are considered background influences rather than direct causes of waste generation.

Regarding Hypothesis H2, the cluster analysis identifies two statistically distinct groups of EU member states based on joint patterns of sectoral food waste and macroeconomic indicators. The robustness of the cluster solution lies in its ability to summarize structural contrasts across countries, not in identifying homogeneous national models of behavior or governance. These clusters summarize structural contrasts across countries but do not represent homogeneous national models of behavior or governance. Substantial internal heterogeneity within clusters limits the usefulness of rigid typological classifications, echoing concerns raised by Caldeira et al. [6] and Brautigam et al. [39]. The results instead suggest that food waste dynamics emerge from interactions between economic structure, sectoral organization, and measurement practices, without implying uniform behavioral patterns within or across country groups. This reinforces the interpretation that food waste dynamics reflect interactions between economic structure, sectoral organization, and measurement practices, without implying homogeneous behavioral patterns within or across country groups.

This interpretation is consistent with governance-oriented research [54,55], which documents substantial variation in institutional capacity and policy implementation across EU member states. While governance arrangements and regulatory frameworks likely interact with economic conditions to influence food waste outcomes, these dimensions are not directly captured by the statistical clustering applied here. Their exclusion underscores the structural, rather than institutional or behavioral, nature of the identified groupings and points to the need for complementary analytical approaches.

From a policy perspective, these findings indicate that food waste reduction strategies should be adapted to sector-specific structural conditions. Interventions targeting manufacturing and distribution may benefit from improvements in logistics, technology, and supply-chain coordination, while household- and consumption-related waste requires complementary approaches beyond macroeconomic instruments. This differentiated perspective is consistent with calls for empirically grounded and context-sensitive policy design [22,23].

These findings highlight that macroeconomic impacts on food waste vary by sector and should not be applied uniformly across all stages of the food supply chain. This is especially true for primary production and households, where non-economic and measurement-related factors are more influential.

This paper interprets the results as system-level structural associations derived from aggregate and sectoral data, and does not infer individual behavioral mechanisms beyond those documented in the existing literature. Pandemic-related effects are treated as contextual influences that may have amplified or dampened sector-specific waste levels. At the same time, the reported results are interpreted as structural associations derived from harmonized cross-country data rather than as direct estimates of the COVID-19 impact.

### 5.1. Theoretical Implications

This study contributes to the theoretical understanding of food waste by clarifying the scope and limits of macroeconomic explanations across different stages of the food supply chain. By situating food waste within a structural economic framework, the analysis shows that income-related indicators are most relevant for explaining waste generated at intermediate stages of the supply chain, where production scale and organizational efficiency are central.

At the same time, the limited role of price dynamics and the weak alignment of household and primary production waste with macroeconomic factors highlight the multidimensional nature of food waste. These findings support theoretical perspectives that emphasize the interaction between economic structures, sectoral organization, and contextual factors, rather than reductionist explanations based solely on income or prices.

The conceptual contribution of the study lies in delineating the boundary conditions of macroeconomic explanations of food waste. While economic abundance shapes system-level waste patterns, its influence is mediated by sector-specific characteristics, institutional contexts, and measurement constraints. This reinforces the need for theoretical models that accommodate structural heterogeneity and acknowledge the limits of macro-level indicators in explaining food waste outcomes.

### 5.2. Practical Implications

The results offer several implications for food waste policy in the European Union. The uneven influence of macroeconomic conditions across sectors suggests that policy interventions should not rely on uniform assumptions about consumer or producer behavior. Instead, strategies should be tailored to the structural characteristics of specific supply-chain stages.

Although the cluster analysis does not yield uniform country typologies, it provides a structural framework for identifying shared economic and sectoral conditions under which food waste is generated. This approach supports the development of differentiated policy priorities, such as investments in supply-chain efficiency in manufacturing and retail, or targeted measures to address consumption-related waste.

At the same time, the substantial heterogeneity observed within clusters implies that policy instruments must remain adaptable to national and local contexts. Structural clustering thus supports a balanced policy framework combining EU-level coordination with country-specific implementation, enhancing the relevance and effectiveness of food waste reduction strategies.

### 5.3. Limitations and Future Research Directions

Several limitations of this study should be acknowledged, which also point to important directions for future research. First, the empirical analysis relies exclusively on secondary data provided by Eurostat. Although these data are harmonized and represent the most comprehensive and policy-relevant source currently available for comparative analysis across EU member states, they remain subject to differences in national data collection practices, estimation techniques, and sectoral reporting standards. Such methodological heterogeneity may affect the precision and cross-country comparability of food waste estimates, particularly for sectors where measurement relies on indirect methods or model-based approximations.

Second, the analytical framework focuses on macroeconomic and sectoral indicators and does not explicitly incorporate behavioral, institutional, or governance-related variables. Food waste generation—especially at the household and food service levels—is influenced by consumer routines, food literacy, social norms, regulatory enforcement, and policy implementation capacity. The absence of these dimensions limits the model’s ability to capture the full complexity of food waste dynamics. It may partly explain the moderate explanatory power observed in the empirical results. Consequently, the findings should be interpreted as reflecting structural economic associations rather than comprehensive explanations of food waste behavior.

A further limitation concerns the temporal scope of the analysis. The study covers the 2020–2022 period, which does not allow for longitudinal assessment or causal inference. This short time horizon constrains the ability to distinguish between persistent structural drivers and short-term adjustments in food waste generation. While the selected period ensures maximum data completeness and comparability across all EU member states, it limits insight into dynamic processes and long-term trends.

In addition, the analysis period coincided with the COVID-19 pandemic, which was a major external shock that affected economic activity, consumption patterns, and supply-chain organization throughout the European Union. Pandemic-related disruptions may have altered food waste generation, particularly in households, food services, and retail, through mechanisms such as mobility restrictions, shifts toward home consumption, and temporary closures of hospitality services. Although the use of harmonized cross-country data allows the results to be interpreted as structural associations under a standard external shock, the findings may partly reflect temporary pandemic-related adjustments rather than stable long-term relationships.

These limitations suggest several avenues for future research. Extending the analysis to longer time spans would enable the application of panel data techniques, allowing researchers to control for unobserved heterogeneity, examine dynamic relationships, and assess how income and price effects on food waste evolve. As additional harmonized food waste data become available, such approaches could significantly enhance the robustness and explanatory depth of empirical findings.

Future studies could also adopt more sector-specific analytical designs, focusing in greater detail on individual stages of the food supply chain, such as households, manufacturing, or retail and distribution, where the determinants of food waste differ substantially. Finally, mixed-methods approaches that combine quantitative modeling with surveys, interviews, or case studies would allow the integration of behavioral and institutional dimensions into the analysis. Such approaches would contribute to a more comprehensive understanding of food waste generation and support the development of more targeted, context-sensitive, and practical policy interventions.

## 6. Conclusions

This study offers a sector-specific, structurally grounded analysis of food waste across European Union member states, using harmonized macroeconomic and sectoral data. The findings show that macroeconomic indicators are relevant for understanding food waste in certain stages of the food supply chain, particularly manufacturing and distribution, but have limited explanatory power for primary production and household waste.

Rather than reinterpreting the empirical results, the conclusions highlight the implications of this structural heterogeneity for policy and research. Effective food waste reduction requires approaches that align economic structures, supply-chain organization, and institutional capacity, while recognizing the limits of macroeconomic instruments in addressing all forms of waste.

Overall, the study underscores the importance of context-sensitive, sector-specific strategies for reducing food waste in advanced economies. By clarifying where macroeconomic explanations are most relevant and where complementary approaches are needed, the analysis provides a foundation for more targeted and effective food waste policies within the European Union.

## Figures and Tables

**Table 1:** Definition, measurement, and data sources of variables used in the analysis.

Variable	Data	Measures	Sources
PPF	Food waste—Primary production of food—agriculture, fishing, and aquaculture	Kilograms per capita	[70]
MFPB	Food waste—Manufacture of food products and beverages	Kilograms per capita	[70]
RODF	Food waste—Retail and other distribution of food	Kilograms per capita	[70]
RFS	Food waste—Restaurants and food services	Kilograms per capita	[70]
TAH	Food waste—Total activities by households	Kilograms per capita	[70]
GDPpc	Gross domestic product (GDP) per capita	Percentage of EU27 (from 2020) total per capita (based on million purchasing power standards, EU27 from 2020), current prices	[71]
HICP	Harmonized Index of Consumer Prices—Food	Index, 2015 = 100	[72]
AGDIpc	Adjusted gross disposable income of households per capita in PPS	Purchasing power standard (PPS, EU27 from 2020), per inhabitant	[73]

Source: designed by the author using data from Eurostat [70,71,72,73].

**Table 2:** Factor loadings from exploratory factor analysis of sectoral food waste and macroeconomic indicators.

	Factor 1
PPF	0.300
MFPB	0.619
RODF	0.673
RFS	0.334
TAH	0.101
GDPpc	0.503
HICP	−0.552
AGDIpc	0.465

Source: designed by the author using SPSS v.27 (IBM Corporation, Armonk, NY, USA).

**Table 3:** Structural path coefficients from the PLS-SEM model.

	Original Sample	Sample Mean	Standard Deviation	T Statistics	*p* Values
Consumer prices—Food → Food waste	−0.214	−0.206	0.157	1.365	0.173
Gross income → Food waste	0.407	0.414	0.162	2.513	0.012

Source: author’s design with SmartPLS v3.0 (SmartPLS GmbH, Bönningstedt, Germany).

## Data Availability

The original contributions presented in the study are included in the article. Further inquiries can be directed to the corresponding author.

## Abbreviations

The following abbreviations are used in this manuscript:

EU	European Union
PPF	Food waste—Primary production of food—agriculture, fishing, and aquaculture
MFPB	Food waste—Manufacture of food products and beverages
RODF	Food waste—Retail and other distribution of food
RFS	Food waste—Restaurants and food services
TAH	Food waste—Total activities by households
GDPpc	Gross domestic product (GDP) per capita
HICP	Harmonized Index of Consumer Prices—Food
AGDIpc	Adjusted gross disposable income of households per capita in PPS
SEM	Structural equation modeling
KMO	Kaiser–Meyer–Olkin
CR	Composite reliability
SRMR	Standardized root mean squared residual
d_ULS	unweighted least squares discrepancy
d_G	geodesic discrepancy

## Appendix A

**Table A1:** Correlation matrix of sectoral food waste indicators and macroeconomic variables.

		PPF	MFPB	RODF	RFS	TAH	GDPpc	HICP	AGDIpc
Correlation	PPF	1.000	0.299	0.596	0.072	0.185	−0.167	−0.110	−0.185
	MFPB	0.299	1.000	0.501	0.077	−0.016	0.260	−0.324	0.281
	RODF	0.596	0.501	1.000	0.305	0.188	0.112	−0.320	0.171
	RFS	0.072	0.077	0.305	1.000	0.166	0.208	−0.247	0.084
	TAH	0.185	−0.016	0.188	0.166	1.000	−0.111	−0.022	−0.004
	GDPpc	−0.167	0.260	0.112	0.208	−0.111	1.000	−0.389	0.710
	HICP	−0.110	−0.324	−0.320	−0.247	−0.022	−0.389	1.000	−0.233
	AGDIpc	−0.185	0.281	0.171	0.084	−0.004	0.710	−0.233	1.000
Sig. (2-tailed)	PPF		0.003	0.000	0.261	0.049	0.068	0.164	0.049
	MFPB	0.003		0.000	0.248	0.445	0.009	0.002	0.006
	RODF	0.000	0.000		0.003	0.046	0.161	0.002	0.064
	RFS	0.261	0.248	0.003		0.069	0.031	0.013	0.227
	TAH	0.049	0.445	0.046	0.069		0.161	0.422	0.486
	GDPpc	0.068	0.009	0.161	0.031	0.161		0.000	0.000
	HICP	0.164	0.002	0.002	0.013	0.422	0.000		0.018
	AGDIpc	0.049	0.006	0.064	0.227	0.486	0.000	0.018	

Source: designed by the author using SPSS v.27 (IBM Corporation, Armonk, NY, USA).

**Table A2:** Descriptive statistics and cluster membership of EU member states (2022).

Countries	PPF	MFPB	RODF	RFS	TAH	GDPpc	HICP	AGDIpc
Czechia	1	15	6	17	61	89.0	121.6	22,724
Slovenia	0	5	7	26	33	88.7	114.95	22,691
Malta	1	14	9	51	88	103.1	121.76	22,852
Spain	16	11	7	6	26	87.7	113.73	22,839
Lithuania	29	14	11	1	86	87.1	127.25	22,182
Portugal	11	6	22	23	123	77.0	108.85	21,611
Ireland	10	44	17	30	42	236.6	93.3	24,345
Subcluster A1 mean	9.71	15.57	11.29	22.00	65.57	109.89	114.49	22,749.14
Bulgaria	10	23	6	15	41	62.1	129.77	15,260
Latvia	14	16	8	13	71	69.0	124.71	16,475
Croatia	10	2	1	4	55	71.6	114.85	18,333
Romania	32	20	2	29	99	73.2	124.75	18,238
Greece	25	44	18	23	86	66.7	106.96	17,916
Estonia	16	29	15	10	65	83.7	124.01	19,074
Slovakia	7	26	6	2	65	71.0	121.87	18,814
Hungary	1	14	6	2	60	76.1	136.01	19,536
Poland	20	15	13	7	69	77.9	129.8	20,268
Subcluster A2 mean	15.00	21.00	8.33	11.67	67.89	72.37	123.64	18,212.67
Cluster A mean	12.69	18.63	9.63	16.19	66.88	88.78	119.64	20,197.38
Germany	2	19	9	24	75	120.1	117.8	34,098
Luxembourg	12	18	13	15	65	247.9	114.49	37,206
Austria	1	23	9	28	70	122.9	110.62	31,052
Netherlands	18	50	9	5	48	133.8	114.09	30,716
Belgium	3	63	11	10	64	118.2	109.48	30,076
Finland	5	25	10	15	55	106.4	104.77	27,010
Sweden	9	29	10	14	56	113.7	113.65	27,480
Cyprus	52	72	60	33	77	94.3	105.95	26,394
Denmark	20	118	17	13	86	133.6	109.8	26,612
Italy	11	9	11	8	100	97.6	110.3	25,795
France	17	35	12	16	58	97.2	111.10	28,594
Cluster B mean	13.64	41.91	15.55	16.45	68.55	125.97	111.10	29,548.45
Eu Mean	13.07	28.11	12.04	16.30	67.56	103.93	116.16	24,007.07

Source: author’s design with SPSS v.27 (IBM Corporation, Armonk, NY, USA).

## References

[1] Cattaneo A., Sánchez M.V., Torero M., Vos R. (2021). Reducing food loss and waste: Five challenges for policy and research. Food Policy.

[2] FAO (2019). Moving Forward on Food Loss and Waste Reduction: The State of Food and Agriculture 2019.

[3] Muñoz-Torres M.J., Ferrero-Ferrero I., Gisbert-Navarro J.V., Rivera-Lirio J.M. (2025). Environmental assessment of food loss and waste prevention and reduction solutions: Navigating the complexity of integrating stakeholders’ decisions. Environ. Impact Assess. Rev..

[4] Fabi C., Cachia F., Conforti P., English A., Rosero Moncayo J. (2021). Improving data on food losses and waste: From theory to practice. Food Policy.

[5] Scherhaufer S., Moates G., Hartikainen H., Waldron K., Obersteiner G. (2018). Environmental impacts of food waste in Europe. Waste Manag..

[6] Caldeira C., De Laurentiis V., Ghose A., Corrado S., Sala S. (2021). Grown and thrown: Exploring approaches to estimate food waste in EU countries. Resour. Conserv. Recycl..

[7] Canali M., Amani P., Aramyan L., Gheoldus M., Moates G., Östergren K., Silvennoinen K., Waldron K., Vittuari M. (2017). Food Waste Drivers in Europe, from Identification to Possible Interventions. Sustainability.

[8] Philippidis G., Sartori M., Ferrari E., M’Barek R. (2019). Waste not, want not: A bio-economic impact assessment of household food waste reductions in the EU. Resour. Conserv. Recycl..

[9] Gencia A.D., Balan I.M. (2024). Reevaluating Economic Drivers of Household Food Waste: Insights, Tools, and Implications Based on European GDP Correlations. Sustainability.

[10] De Laurentiis V., Corrado S., Sala S. (2018). Quantifying Household Waste of Fresh Fruit and Vegetables in the EU. Waste Manag..

[11] Corrado S., Sala S. (2018). Food waste accounting along global and European food supply chains: State of the art and outlook. Waste Manag..

[12] Smith T.A., Landry C.E. (2021). Household food waste and inefficiencies in food production. Am. J. Agric. Econ..

[13] Bishop M., Megicks P. (2019). “Waste not, want not!”: Qualitative insights into consumer food waste behaviour. Waste Manag. Environ..

[14] Akhter S., Rather M.I., Zargar U.R. (2024). Understanding the food waste behaviour in university students: An application of the theory of planned behaviour. J. Clean. Prod..

[15] Attiq S., Habib M.D., Kaur P., Hasni M.J.S., Dhir A. (2021). Drivers of food waste reduction behaviour in the household context. Food Qual. Prefer..

[16] Stancu V., Haugaard P., Lähteenmäki L. (2016). Determinants of Consumer Food Waste Behavior: Two Routes to Food Waste. Appetite.

[17] Young C.W., Russell S.V., Robinson C.A., Chintakayala P.K. (2018). Sustainable retailing—Influencing consumer behaviour on food waste. Bus. Strategy Environ..

[18] Bocean C.G. (2024). A Longitudinal Analysis of the Impact of Digital Technologies on Sustainable Food Production and Consumption in the European Union. Foods.

[19] Adaryani R.L., Palouj M., Gholami H., Baghestany A.A., Damirchi M.J., Dadar M., Seifollahi N. (2025). Predicting household food waste behavior: Bringing food literacy and purchasing power into the theory of planned behavior. J. Retail. Consum. Serv..

[20] Szulecka J., Bradshaw C., Principato L. (2024). Food waste governance architectures in Europe: Actors, steering modes, and harmonization trends. Glob. Chall..

[21] Giordano C., Falasconi L., Cicatiello C., Pancino B. (2019). The role of food waste hierarchy in addressing policy and research: A comparative analysis. J. Clean. Prod..

[22] Wang Y., Yuan Z., Tang Y. (2021). Enhancing food security and environmental sustainability: A critical review of food loss and waste management. Resour. Environ. Sustain..

[23] Zaharia A., Diaconeasa M.-C., Maehle N., Szolnoki G., Capitello R. (2021). Developing Sustainable Food Systems in Europe: National Policies and Stakeholder Perspectives in a Four-Country Analysis. Int. J. Environ. Res. Public Health.

[24] European Commission (2014). Towards a Circular Economy: A Zero Waste Programme for Europe.

[25] European Commission (2019). EU Platform on Food Losses and Food Waste.

[26] Economou F., Chatziparaskeva G., Papamichael I., Loizia P., Voukkali I., Navarro-Pedreño J., Klontza E., Lekkas D.F., Naddeo V., Zorpas A.A. (2024). The concepts of food waste, food loss prevention, and measuring tools. Waste Manag. Res..

[27] Aitsidou V., Michailidou E., Loizou E., Tsantopoulos G., Michailidis A. (2024). Focus Group Discussions on Food Waste: Insights into rural and urban households in Greece. Sustainability.

[28] FAO (2014). Food Wastage Footprint: Impacts on Natural Resources.

[29] Stenmarck Å., Jensen C., Quested T., Moates G. (2016). Estimates of European Food Waste Levels.

[30] Rutten M., Nowicki P., Bogaardt M.-J., Aramyan L. (2013). Reducing Food Waste by Households and in Retail in the EU: A Prioritisation Using Economic, Land Use and Food Security Impacts.

[31] Rutten M., Shutes L., Meijerink G. (2013). Sit down at the ball game: How trade barriers make the world less food secure. Food Policy.

[32] Campoy-Muñoz P., Cardenete M.A., Delgado M.C. (2017). Economic impact assessment of food waste reduction on European countries through social accounting matrices. Resour. Conserv. Recycl.

[33] Bocean C.G. (2024). A Cross-Sectional Analysis of the Relationship between Digital Technology Use and Agricultural Productivity in EU Countries. Agriculture.

[34] Verghese K., Lewis H., Lockrey S., Williams H. (2015). Packaging’s role in minimizing food loss and waste across the supply chain. Packag. Technol. Sci..

[35] Williams H., Lindström A., Trischler J., Wikström F., Rowe Z. (2020). Avoiding food becoming waste in households—The role of packaging in consumers’ practices. J. Clean. Prod..

[36] Ajzen I. (1991). The Theory of Planned Behavior. Organ. Behav. Hum. Decis. Process..

[37] Graham-Rowe E., Jessop D.C., Sparks P. (2014). Identifying motivations and barriers to minimizing household food waste. Resour. Conserv. Recycl..

[38] Kansal M., Mitsis A., Haque A., Ananda J., Pearson D. (2025). Understanding household food waste using a psychographic segmentation. Australas. J. Environ. Manag..

[39] Brautigam K.-R., Jörissen J., Priefer C. (2014). The extent of food waste generation across the EU-27: Different calculation methods and the reliability of their results. Waste Manag. Res..

[40] Garcia-Garcia G., Woolley E., Rahimifard S., Colwill J., White R., Needham L. (2017). A methodology for sustainable management of food waste. Waste Biomass Valorization.

[41] Calabró G., Vieri S. (2024). Food waste and the EU target: Effects on the agri-food systems’ sustainability. J. Foodserv. Bus. Res..

[42] European Commission (2018). A Sustainable Bioeconomy for Europe: Strengthening the Connection Between Economy, Society and the Environment.

[43] Secondi L., Principato L., Laureti T. (2015). Household food waste behaviour in EU-27 countries: A multilevel analysis. Food Policy.

[44] Setti M., Falasconi L., Segrè A., Cusano I., Vittuari M. (2016). Italian consumers’ income and food waste behavior. Br. Food J..

[45] Thyberg K.L., Tonjes D.J. (2016). Drivers of food waste and their implications for sustainable policy development. Resour. Conserv. Recycl..

[46] Visschers V.H.M., Wickli N., Siegrist M. (2016). Sorting out food waste behaviour: A survey on the motivators and barriers of self-reported amounts of food waste in households. J. Environ. Psychol..

[47] Bajželj B., Quested T.E., Röös E., Swannell R.P.J. (2020). The role of reducing food waste for resilient food systems. Ecosyst. Serv..

[48] Quested T., Parry A., Easteal S., Swannell R. (2011). Food and drink waste from households in the UK. Nutr. Bull..

[49] Schanes K., Dobernig K., Gözet B. (2018). Food waste matters—A systematic review of household food waste practices and their policy implications. J. Clean. Prod..

[50] Stefan V., van Herpen E., Tudoran A.A., Lähteenmäki L. (2013). Avoiding food waste by Romanian consumers: The importance of planning and shopping routines. Food Qual. Prefer..

[51] Mourad M. (2016). Recycling, recovering and preventing “food waste”: Competing solutions for food systems sustainability in the United States and France. J. Clean. Prod..

[52] Azzurro P., Gaiani S., Vittuari M. (2016). Italy—Country Report on National Food Waste Policy.

[53] Bradshaw C. (2020). England’s fresh approach to food waste: Problem frames in the Resources and Waste Strategy. Leg. Stud..

[54] Szulecka J., Strøm-Andersen N. (2022). Norway’s Food Waste Reduction Governance: From Industry Self-Regulation to Governmental Regulation?. Scand. Political Stud..

[55] Tosun J., Saad E.L., Glückler J., Irigoyen Rios A., Lehmann R. (2023). Country-Specific Participation Patterns in Transnational Governance Initiatives on Sustainability: Preliminary Insights and Research Agenda. Glob. Chall..

[56] Gumbert T. (2022). Responsibility in Environmental Governance: Unwrapping the Global Food Waste Dilemma.

[57] Porter S.D. (2020). Food waste in the UK and EU: A policy and practice perspective. Routledge Handbook of Food Waste.

[58] Papargyropoulou E., Lozano R., Steinberger J.K., Wright N., bin Ujang Z. (2014). The food waste hierarchy as a framework for the management of food surplus and food waste. J. Clean. Prod..

[59] European Commission (2025). Food Waste.

[60] Jain A.K. (2010). Data clustering: 50 years beyond K-means. Pattern Recognit. Lett..

[61] Xu D., Tian Y. (2015). A comprehensive survey of clustering algorithms. Ann. Data Sci..

[62] Caldeira C., De Laurentiis V., Corrado S., van Holsteijn F., Sala S. (2019). Quantification of Food Waste per Product Group along the Food Supply Chain in the EU. Resour. Conserv. Recycl..

[63] Stecuła K., Wolniak R., Aydın B. (2024). Technology Development in Online Grocery Shopping—From Shopping Services to Virtual Reality, Metaverse, and Smart Devices: A Review. Foods.

[64] Priefer C., Jörissen J., Bräutigam K.-R. (2016). Food waste prevention in Europe—A cause-driven approach to identify the most relevant leverage points for action. Resour. Conserv. Recycl..

[65] Göbel C., Langen N., Blumenthal A., Teitscheid P., Ritter G. (2015). Cutting Food Waste through Cooperation along the Food Supply Chain. Sustainability.

[66] Närvänen E., Mesiranta N., Mattila M., Heikkinen A. (2020). Food Waste Management: Solving the Wicked Problem.

[67] Magalhães V.S.M., Ferreira L.M.D.F., Silva C. (2021). Using a methodological approach to model causes of food loss and waste in fruit and vegetable supply chains. J. Clean. Prod..

[68] Vanham D., Bouraoui F., Leip A., Grizzetti B., Bidoglio G. (2015). Lost water and nitrogen resources due to EU consumer food waste. Environ. Res. Lett..

[69] Springmann M., Clark M., Mason-D’Croz D., Wiebe K., Bodirsky B.L., Lassaletta L., de Vries W., Vermeulen S.J., Herrero M., Carlson K.M. (2018). Options for keeping the food system within environmental limits. Nature.

[70] Eurostat Food Waste and Food Waste Prevention by NACE Rev. 2 Activity. https://ec.europa.eu/eurostat/databrowser/view/env_wasfw__custom_18351658/default/table?page=time:2022.

[71] Eurostat Gross Domestic Product (GDP) and Main Components Per Capita. https://ec.europa.eu/eurostat/databrowser/view/nama_10_pc/default/table?lang=en.

[72] Eurostat Harmonised Index of Consumer Prices—Food. https://ec.europa.eu/eurostat/databrowser/view/prc_fsc_idx__custom_18351959/default/table.

[73] Eurostat Adjusted Gross Disposable Income of Households Per Capita in PPS. https://ec.europa.eu/eurostat/databrowser/view/tec00113/default/table?lang=en&category=t_na10.t_nasa_10.

[74] Field A. (2024). Discovering Statistics Using IBM SPSS Statistics.

[75] Aldrich J.O. (2018). Using IBM SPSS Statistics: An Interactive Hands-On Approach.

[76] Penn State, Eberly College of Science Agglomerative Hierarchical Clustering. https://online.stat.psu.edu/stat505/lesson/14/14.4.

[77] Bartelings H., Philippidis G. (2024). A novel macroeconomic modelling assessment of food loss and waste in the EU: An application to the sustainable development goal of halving household food waste. Sustain. Prod. Consum..

